# Monocytes from patients with Primary Ciliary Dyskinesia show enhanced inflammatory properties and produce higher levels of pro-inflammatory cytokines

**DOI:** 10.1038/s41598-017-15027-y

**Published:** 2017-11-07

**Authors:** M. Cockx, M. Gouwy, P. Ruytinx, I. Lodewijckx, A. Van Hout, S. Knoops, N. Pörtner, I. Ronsse, L. Vanbrabant, V. Godding, K. De Boeck, J. Van Damme, M. Boon, S. Struyf

**Affiliations:** 1 Department of Microbiology and Immunology, Rega Institute for Medical Research, Laboratory of Molecular Immunology, University of Leuven, Leuven, Belgium; 2 Unité de Pneumologie Pédiatrique et Mucoviscidose, Clinique Universitaire Saint-Luc UCL Bruxelles, Bruxelles, Belgium; 3 Pediatric Pulmonology and Cystic Fibrosis Unit, Department of Pediatrics, University hospitals Leuven, Leuven, Belgium

## Abstract

Patients with Primary Ciliary Dyskinesia (PCD) suffer from recurrent upper and lower airway infections due to defects in the cilia present on the respiratory epithelium. Since chronic inflammatory conditions can cause changes in innate immune responses, we investigated whether monocytes isolated from the peripheral blood of pediatric PCD patients respond differently to inflammatory stimuli, compared to monocytes from healthy children and adults. The receptor for C5a (C5aR) was upregulated in PCD, whereas expression levels of the leukocyte chemoattractant receptors CCR1, CCR2, CCR5, BLT1 and FPR1 on PCD monocytes were similar to those on monocytes from healthy individuals. Also *in vitro* migration of PCD monocytes towards the ligands of those receptors (CCL2, fMLP, C5a and LTB4) was normal. Compared to healthy children, PCD patients had a higher percentage of the non-classic monocyte subset (CD14+CD16++) in circulation. Finally, PCD monocytes produced higher levels of pro-inflammatory cytokines (IL-1β and TNF-α) and chemokines (CCL3, CCL5, CCL18 and CCL22) in response to LPS, peptidoglycan and/or dsRNA stimulation. These data suggest that monocytes might exacerbate inflammatory reactions in PCD patients and might maintain a positive feedback-loop feeding the inflammatory process.

## Introduction

Primary Ciliary Dyskinesia (PCD) is an autosomal inherited recessive disorder caused by mutations leading to structural and/or functional defects of motile cilia. It is a rare disease with an incidence of 1/10,000–1/20,000 individuals^1–3^. In the respiratory tract, motile cilia are essential in protecting the lungs from injury and infection by upward transport of the mucus, in which harmful microorganisms and particles are captured. This process is called mucociliary clearance and its deficiency, due to ineffective movement of the motile cilia, causes recurrent infections of the upper and lower airways in PCD patients^4^.

To date, about 35 genes have been linked to PCD, mostly leading to loss or abnormal functioning of the energy-producing ciliary dynein proteins^5–7^. About 30 percent of the PCD patients, however, carry a yet unknown gene mutation. The genetic heterogeneity is linked to the complex structure of motile cilia, consisting of more than 250 different proteins^8^. This results in heterogeneity of symptoms and disease severity, which challenges both diagnosis and treatment of PCD. Management of PCD aims to control the recurrent airway infections and maintain lung function by administering antibiotics and stimulating airway clearance, respectively^9^. To date, few studies have been performed to reveal the pathophysiologic processes in PCD other than ciliary motion that may contribute to the disease.

The aim of this study is to unravel the role of the innate immune system as a possible factor of heterogeneity in disease severity in PCD. Indeed, in cystic fibrosis (CF), another lung disease characterized by impaired mucociliary clearance, several aspects of the innate immune responses are abnormal^10^. The CFTR gene affected in CF is expressed in neutrophils and in monocytes and leads to aberrant leukocyte responses, but also secondary effects of the chronic inflammation on innate leukocyte function have been demonstrated. PCD and CF are both characterized by a prominent neutrophilic lung infiltrate, which might be caused by overproduction of the chemokine CXCL8^11,12^. Despite the high neutrophil count, CF and PCD patients suffer from recurrent bacterial airway infections. To clear those infections, monocytes are also crucial as they are key players in detecting pathogens and activating other blood leukocytes (e.g. neutrophils) by secreting cytokines and chemokines^13–15^. Chemokines or chemotactic cytokines are first secreted by resident cells at the site of infection, to attract additional monocytes and other phagocytes to combat the infection. Specific chemokines (CCL and CXCL), recognized by monocytes via their corresponding chemoattractant receptors (CCR and CXCR), generate a chemokine concentration gradient along which monocytes migrate to reach the site of infection. Other chemoattractants recognizing different chemoattractant receptors, such as N-formylmethionine-leucyl-phenylalanine (fMLP), leukotriene B4 (LTB4) and complement component 5a (C5a) assist in guiding and activating monocytes. Chemoattractants activate immune cells by binding to their corresponding chemoattractant receptors. These receptors are 7-transmembrane G protein-coupled receptors (GPCR) and altered expression has been implicated in many inflammatory diseases, e.g. rheumatoid arthritis and inflammatory bowel disease^16^. Chemoattractants are thus essential for a properly functioning immune system^17,18^. More recently, monocytes have been recognized as a phenotypically and functionally heterogeneous population^15^. The monocyte subtypes can be differentiated from each other according to their CD14 and CD16 expression. In some inflammatory disorders, an altered ratio of classic (CD14++CD16−), intermediate (CD14++CD16+) and non-classic monocytes (CD14+CD16++) in the blood circulation has been associated with disease progression^19,20^, but no information is available on the relative abundance of the different monocyte subsets in PCD.

Therefore, we decided to investigate whether monocytes from PCD patients are affected functionally, either through the PCD gene defect or through the chronic inflammatory milieu. We thus first measured the chemoattractant receptor expression on monocytes of PCD patients and their capability to respond to their ligands CCL2, LTB4, fMLP and C5a. Further, we examined the phagocytic capacity and the regulation of adhesion molecules on monocytes of PCD patients. We also defined the number of classic, intermediate and non-classic monocytes in the blood circulation of PCD patients *versus* healthy controls. Finally, we determined levels of several cytokines produced by activated PCD monocytes.

## Results

### Patient characteristics

Thirty six patients with PCD (range 2–26 years, median age 13 years; for details see Table 1) were enrolled in this study between June 2012 and November 2016 at the University hospitals of Leuven. Patients were only included when they were clinically stable (no change in cough or sputum, no fever, no change in therapy for a period of at least 2 weeks, change in forced expiratory volume in 1 second (FEV1) < 10% since the last measurement). Diagnosis was made based on a combination of tests: electron microscopy to detect ultrastructural cilium changes, video-microscopy analysis to evaluate ciliary motility, nasal nitric oxide measurements and genetic analysis. Electron microscopy of the cilia showed abnormalities in 20 and normal ultrastructure in 16 patients. All patients displayed abnormal ciliary activity after cell culture of nasal or bronchial biopsies. Genetic analysis confirmed disease causing mutations in 24 patients, did not provide evidence for mutations in 7 patients and was not performed in 5 patients. Because previous studies observed differences in chemotaxis of monocytes between infants (younger than 2 years) and adults^21^, we also included a pediatric control group in these experiments. The adult controls were indispensable as reference for both PCD and healthy children, because testing of healthy children and PCD patients on the same day was impossible. We rather preferred this approach than performing functional assays with frozen cells.

**Table 1:** Patient characteristics.

	PCD	Healthy adult controls	Healthy pediatric controls
**Female/Male No.**	18/18	14/5	15/6
**Age, median (range) in years**	13 (2–26)	28.3 (23–41)	9.5 (4–18)
**Ultrastructural defect**			
Normal ultrastructure	16/36		
ODA	16/36		
IDA	1/36		
CP	3/36		
Ciliary aplasia	1/36		
**Clinical features**			
*Situs inversus*	13/36		
Bronchiectasis	22/36		
FVC, median (range) (%)	96.5 (65–122)		
FEV_1_, median (range) (%)	91 (38–113)		
**Laboratory features**, **median (range)**			
WBC count (/µl)	7300 (4640–17240)		
CRP (mg/dl)	1.1 ( < 0.3–4.9)		
**Sputum bacteria**			
*P. aeruginosa*	3/36		
*H. influenzae*	7/36		
*S. aureus*	6/36		
*S. pneumoniae*	1/36		
*M. catarrhalis*	2/36		
**Treatment**			
Oral antibiotics	5/36		
Intravenous immunoglobulins	2/36		
Subcutaneous immunoglobulins	1/36		
Azithromycin	10/36		
Inhaled steroids	13/36		

CP: central pair abnormalities; CRP: C-reactive protein; FEV_1_: forced expiratory volume after 1 second; FVC: forced vital capacity; IDA: inner dynein arm deficiency; ODA: outer dynein arm deficiency; WBC: white blood cells.

### Monocytes of PCD patients show increased C5aR expression

**Figure 1:**
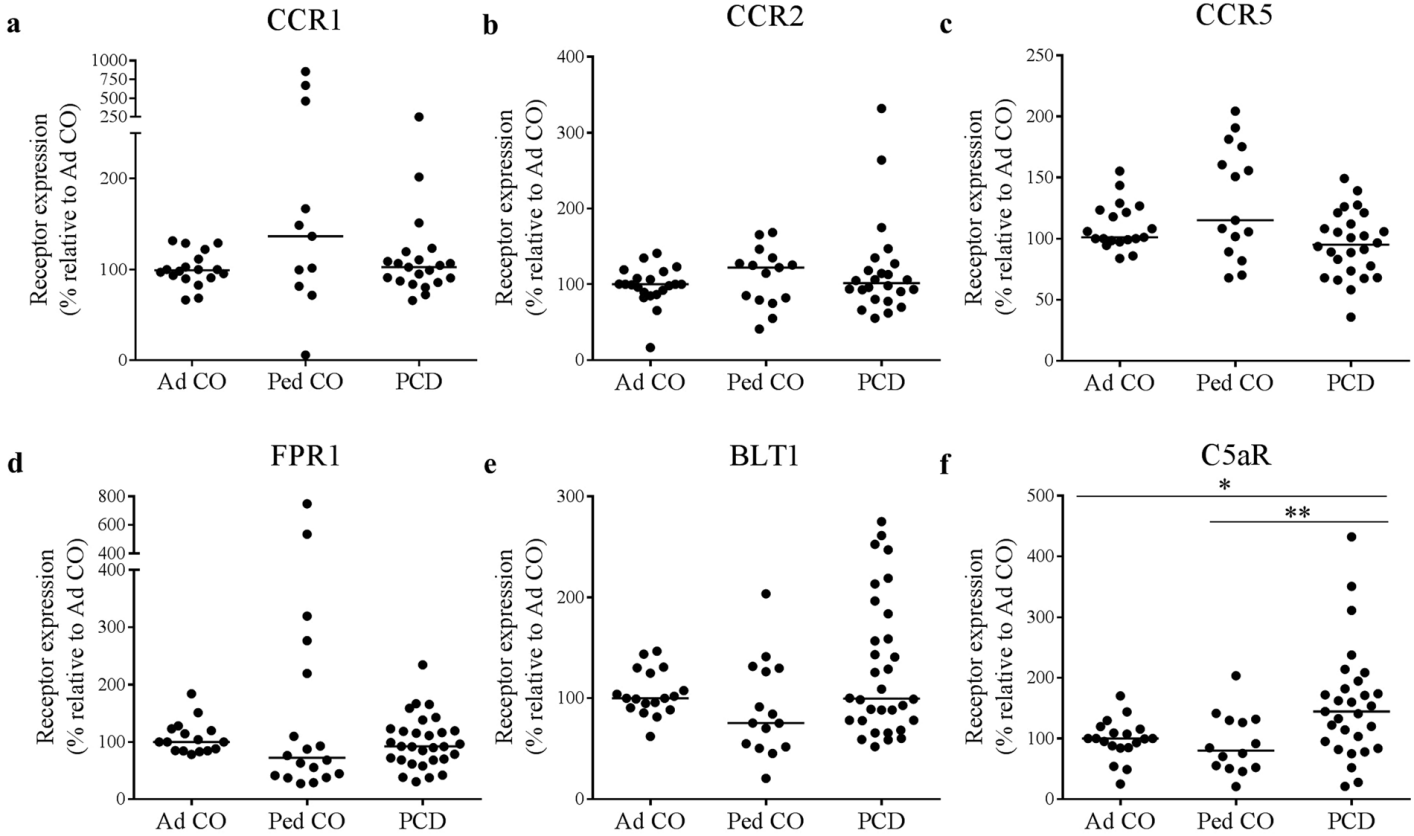
Chemoattractant receptor expression levels on monocytes of PCD patients and healthy controls. Expression levels of (**a**) CCR1, (**b**) CCR2, (**c**) CCR5, (**d**) FPR1, (**e**) BLT1 and (**f**) C5aR on monocytes of PCD patients (PCD), age-matched healthy pediatric controls (Ped CO) and healthy adult controls (Ad CO) were measured by flow cytometry. The chemoattractant receptor expression levels (mean fluorescence intensity, MFI) were normalized to the levels on monocytes of the reference Ad CO (%). Each dot represents the normalized MFI of one patient or healthy control (*p < 0.05, **p < 0.01; Mann Whitney U-test).

We investigated the expression of several GPCR chemoattractant receptors on monocytes of PCD patients (PCD), age-matched pediatric controls (Ped CO) and healthy adult controls (Ad CO). The expression of CCR1, CCR2, CCR5, FPR1 (fMLP receptor), BLT1 (the LTB4 receptor) and C5aR (C5a receptor) on adult and pediatric controls was similar (Fig. 1). C5aR expression was significantly higher on monocytes of PCD patients compared to both control groups (p < 0.05, p < 0.01) (Fig. 1f). The key monocyte chemokine receptors (CCR1 and CCR2) were expressed at a similar level on monocytes from PCD patients and healthy adults/children (Fig. 1a,b). Finally, the expression levels of CCR5, FPR1 and BLT1 were also normal on PCD monocytes (Fig. 1c–e).

### Altered chemoattractant receptor expression on monocytes from PCD patients does not influence the migration capacity

**Figure 2:**
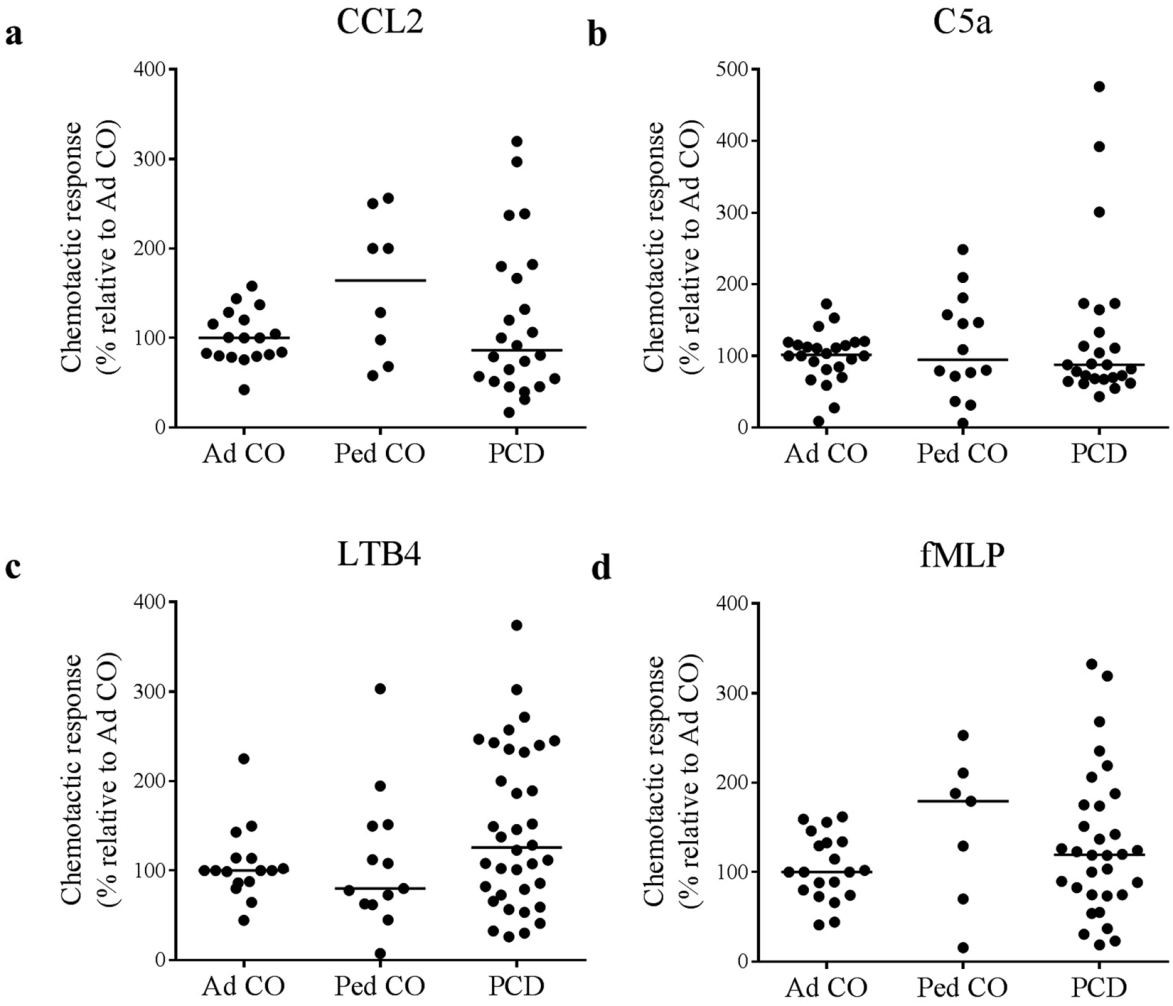
Monocytes from PCD patients and healthy controls are equally responsive in migration assays. Chemotactic responses of monocytes from PCD patients (PCD), age-matched healthy pediatric controls (Ped CO) and healthy adult controls (Ad CO) to (**a**) CCL2 (10 ng/ml), (**b**) C5a (10 ng/ml), (**c**) LTB4 (10-8 M) and (**d**) fMLP (10-8 M) were assessed in the Boyden chamber chemotaxis assay. The chemotactic index (CI; average number of migrated cells in response to the chemoattractant divided by the average number of spontaneously migrated cells) was used to express the chemotactic response. The CIs were normalized to the CI of the reference Ad CO (%). No statistical differences in chemotactic responses of monocytes from PCD patients and monocytes of healthy controls were detected (Mann Whitney U-test).

We next investigated whether the migration of monocytes from PCD patients is affected (Fig. 2). We did not observe significant differences between migration of cells from Ped CO and Ad CO. We observed that the altered C5aR expression on monocytes from PCD patients had no effect on the migration capacity to C5a (Fig. 2b). Migration towards the CCR1/CCR5 ligand CCL3L1 was tested on cells from only a few patients (n = 7) and did also not differ from healthy adults/children (data not shown). The chemotactic response to CCL2, LTB4 and fMLP was similar for monocytes of PCD patients and healthy individuals (Fig. 2a,c,d).

### Circulating monocytes from PCD patients show normal CD62L, but altered CD15 expression upon stimulation with fMLP

**Figure 3:**
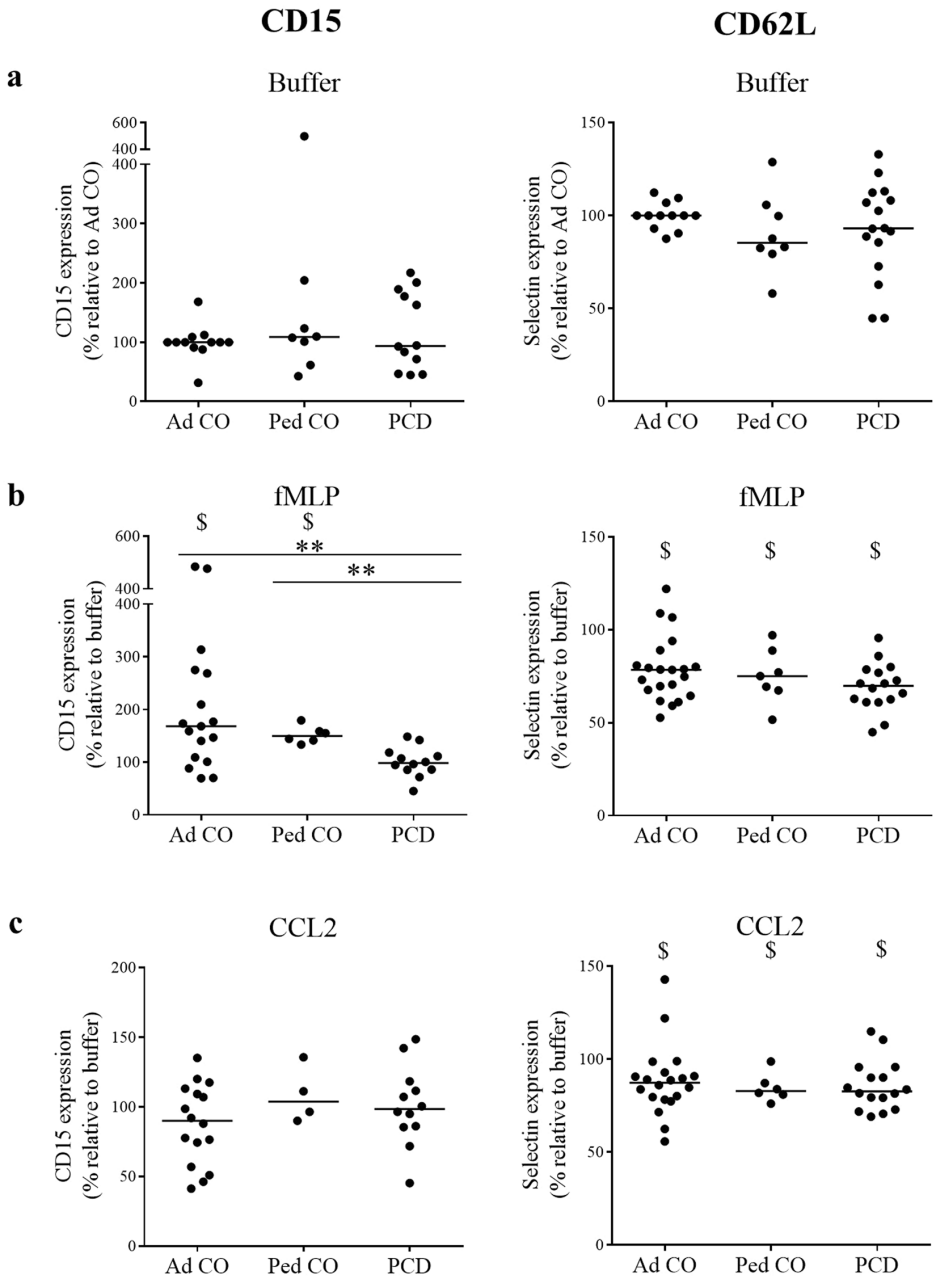
Circulating monocytes of PCD patients show reduced CD15 upregulation upon fMLP stimulation. The expression levels of the selectin ligand CD15 and the selectin CD62L on monocytes of PCD patients (PCD), age-matched healthy pediatric controls (Ped CO) and healthy adult controls (Ad CO) were measured by flow cytometry, either in (**a**) buffer (untreated) or upon (**b**) 10^−8^ M fMLP or (**c**) 300 ng/ml CCL2 stimulation. The expression levels were normalized to the expression level of the reference Ad CO (%). The expression levels in b and c were normalized to the expression levels in buffer conditions. ‘$’Indicate significant differences in expression levels of the adhesion molecule on the monocytes in response to fMLP or CCL2 stimulation compared to buffer treatment. ‘*’Indicate differences between the PCD patients and a control group (**p < 0.01; Mann Whitney U-test).

To reach the site of infection, leukocytes need to extravagate through the endothelium, a process regulated by successive interactions between adhesion molecules on leukocytes and the blood endothelium. We measured the expression of the selectin CD62L and of CD15 (a Lewis^X^ carbohydrate antigen that binds selectins on endothelium) on monocytes in whole blood by flow cytometry (Fig. 3). Chemoattractant-activation of monocytes leads to upregulation of CD15 and downregulation of CD62L to allow subsequent integrin-mediated firm adhesion on the blood endothelium. fMLP and CCL2 are acknowledged stimuli of leukocyte adhesion to the endothelium^22–26^. First, we analyzed basal expression of those molecules on unstimulated monocytes (Fig. 3a). The expression levels were calculated relative to the levels on the monocytes from the Ad CO. The expression levels of CD15 and CD62L were similar on resting monocytes from healthy adults/children and PCD patients. fMLP caused a more pronounced effect (upregulation of CD15 and downregulation of CD62L) than CCL2 (Fig. 3b,c). Upon fMLP stimulation, we measured significantly less upregulation of CD15 on PCD monocytes compared to both healthy control groups (p < 0.01) (Fig. 3b, CD15). CCL2 stimulation, however, resulted in no significant differences in up- or downregulation of adhesion molecules among the three groups (Fig. 3c). However, it must be noted that CD15 expression on monocytes from healthy individuals was not significantly altered in response to CCL2 stimulation.

### Monocytes from PCD patients have similar phagocytic capacities compared to healthy controls

**Figure 4:**
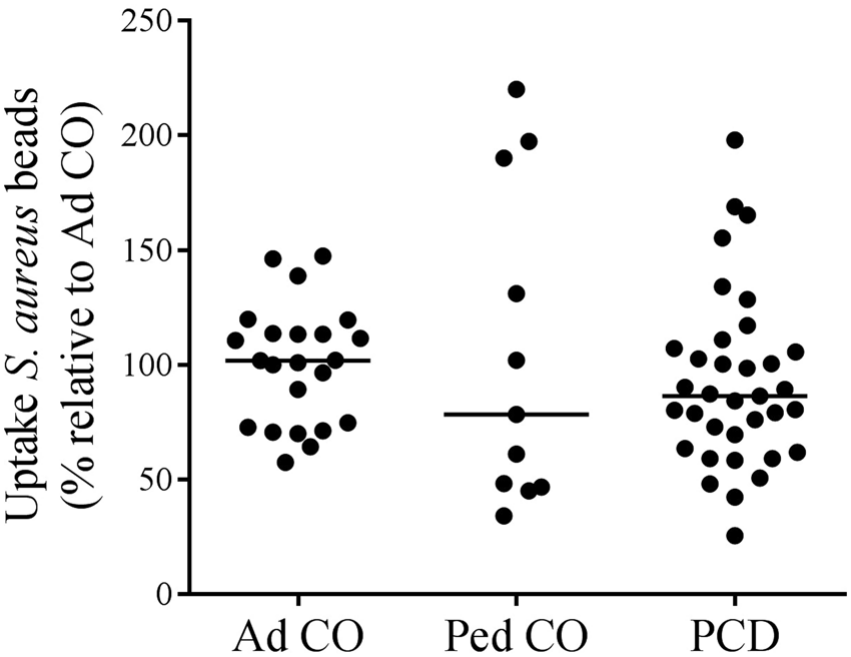
The phagocytic capacity of monocytes from patients with PCD is similar to that of monocytes from healthy controls. The phagocytic capacity of monocytes from PCD patients (PCD), age-matched healthy pediatric controls (Ped CO) and healthy adult controls (Ad CO) was assessed by using *Staphylococcus aureus* labeled beads. The fluorescence intensity of these beads increases when taken up in the acid phagolysosomes of phagocytes. The fluorescence signal of the beads was measured by flow cytometry. The uptake of *S*. *aureus* labeled beads by monocytes from healthy children and PCD patients was expressed relative to the uptake by monocytes of the reference Ad CO (%). No significant difference in phagocytosis was detected between the monocytes of the PCD patients and the healthy controls (Mann Whitney U-test).

One of the main activities executed by monocytes during infection and inflammation is phagocytosis. We tested the phagocytic capacity of monocytes with fluorescent beads coated with *S*. *aureus* (Fig. 4). Monocytes from PCD patients displayed a lower capacity to phagocytose compared to Ad CO (not significant), but we observed similarly reduced phagocytic capacity in Ped CO.

### Higher numbers of non-classic monocytes are present in the circulation of PCD patients compared to age-matched healthy controls

**Figure 5:**
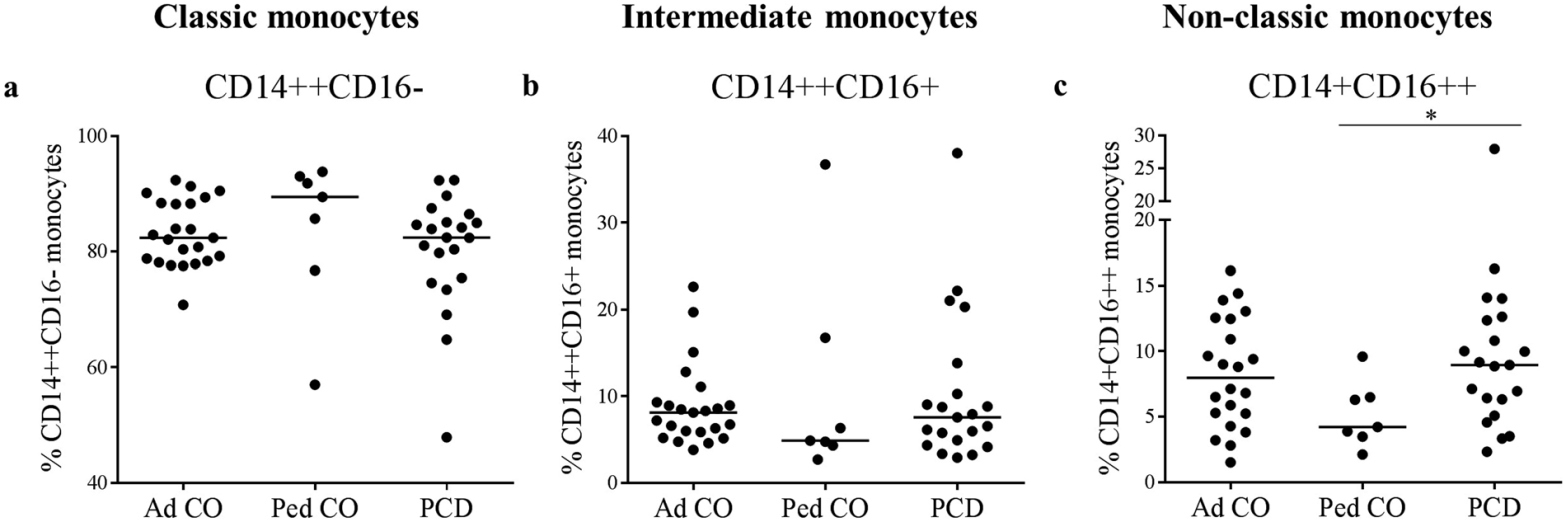
Comparison of the ratio of monocyte subpopulations in the blood circulation of PCD patients and healthy controls. The number of (**a**) classic (CD14++CD16−), (**b**) intermediate (CD14++CD16+) and (**c**) non-classic (CD14+CD16++) monocytes in the blood circulation of PCD patients (PCD), age-matched healthy pediatric controls (Ped CO) and healthy adult controls (Ad CO) was determined by flow cytometry. The proportion of each monocyte subpopulation was calculated by dividing the number of monocytes in one subpopulation by the total number of detected monocytes (*p < 0.05; Mann Whitney U-test).

More recently, different subpopulations in peripheral blood monocytes have been defined^19^. We analyzed by flow cytometry whether a shift between those monocyte subgroups can be observed in PCD patients (Fig. 5). The three groups showed a statistically similar number of CD14++CD16− monocytes, although somewhat higher numbers were detected in Ped CO (Fig. 5a, Ped CO *versus* Ad CO), as already described before^27^. As a consequence, children tend to have lower percentages of circulating non-classic CD14+CD16++ monocytes compared to adults. However, the non-classic CD14+CD16++ monocyte subset was significantly increased in PCD patients compared to Ped CO (p < 0.05) (Fig. 5c).

### PBMCs from PCD patients produce more pro-inflammatory cytokines and chemokines compared to healthy controls

**Figure 6:**
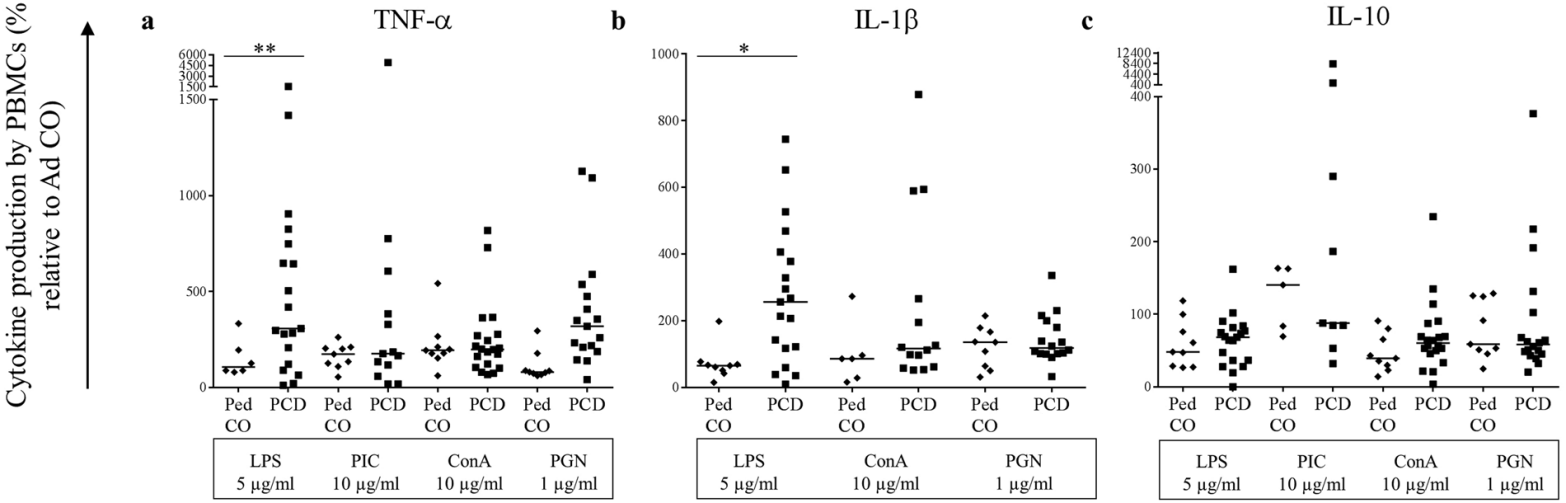
PBMCs from PCD patients produce more pro-inflammatory cytokines (TNF-α and IL-1β) compared to PBMCs from pediatric healthy controls. PBMCs from PCD patients (PCD), age-matched healthy pediatric controls (Ped CO) and healthy adult controls (Ad CO) were cultured and stimulated with LPS (5 µg/ml), dsRNA (PIC; 10 µg/ml), conA (10 µg/ml) or PGN (1 µg/ml) at 37 °C for 24 h. By sandwich ELISAs, (**a**) TNF-α, (**b**) IL-1β and (**c**) IL-10 levels were measured in the supernatants. The cytokine levels are expressed relative to the cytokine levels of the reference Ad CO (equal to 100%, not shown) (*p < 0.05, **p < 0.01; Mann Whitney U-test).

**Figure 7:**
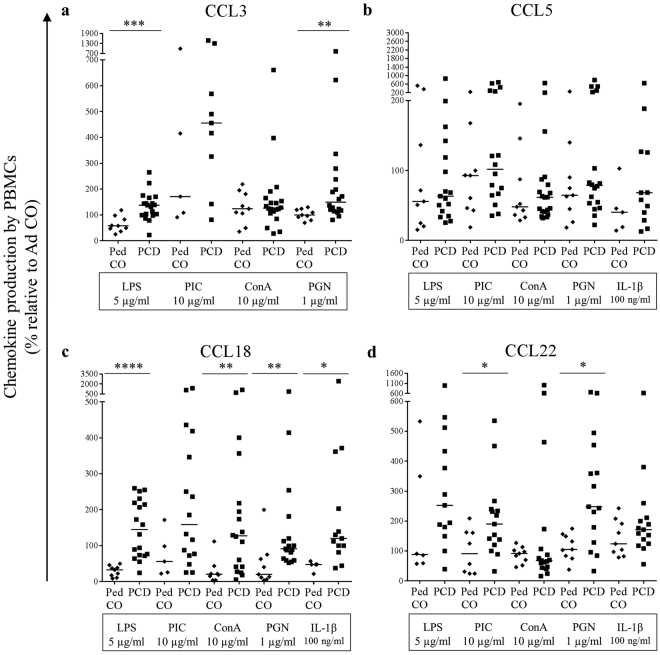
PBMCs from PCD patients produce more pro-inflammatory chemokines (CCL3, CCL18 and CCL22) compared to PBMCs from pediatric healthy controls. PBMCs from PCD patients (PCD), age-matched healthy pediatric controls (Ped CO) and healthy adult controls (Ad CO) were cultured and stimulated with LPS (5 µg/ml), dsRNA (PIC; 10 µg/ml), conA (10 µg/ml), PGN (1 µg/ml) or IL-1β (100 ng/ml) at 37 °C for 24 h. By sandwich ELISAs, (**a**) CCL3, (**b**) CCL5, (**c**) CCL18 and (**d**) CCL22 levels were measured in the supernatants. The cytokine levels are expressed relative to the chemokine levels produced by PBMCs from the reference Ad CO (equal to 100%, not shown). (*p < 0.05, **p < 0.01, ***p < 0.001, ****p < 0.0001; Mann Whitney U-test).

Finally, we investigated the capability of PCD peripheral blood mononuclear cells (PBMCs) to produce cytokines and chemokines to stimulate and attract other immune cells to the site of infection. We stimulated PBMCs from PCD patients, Ped CO and Ad CO with the inflammatory mediators LPS, dsRNA (PIC), conA, PGN and IL-1β. The induced cytokines and chemokines present in the supernatants after 24 h of stimulation were measured by ELISA (Figs 6,7). In response to the TLR4 ligand LPS, PBMCs from PCD patients secreted significant higher levels of TNF-α (p < 0.01) (Fig. 6a), IL-1β (p < 0.05) (Fig. 6b), CCL3 (p < 0.001) (Fig. 7a) and CCL18 (p < 0.0001) (Fig. 7c). We also measured higher levels of CCL22 (Fig. 7d) in response to LPS treatment, but the production was not significantly increased. In response to the TLR3 ligand dsRNA (PIC), which was the weakest inducer, we measured higher CCL22 levels (p < 0.05) (Fig. 7d) in supernatants of PCD PBMCs. Higher CCL5 (Fig. 7b) and CCL18 (p < 0.01) (Fig. 7c) levels were detected in the conditioned media of PCD PBMCs compared to Ped CO cultures upon conA stimulation. Addition of the TLR2 ligand PGN led to elevated production of TNF-α (Fig. 6a), CCL3 (p < 0.01) (Fig. 7a), CCL18 (p < 0.01) (Fig. 7c) and CCL22 (p < 0.05) (Fig. 7d) in PBMC cultures of PCD compared to Ped CO. Finally, upon IL-1β stimulation increased CCL5 (Fig. 7b), CCL18 (p < 0.05) (Fig. 7c) and CCL22 (Fig. 7d) concentrations were measured in supernatants of PBMCs isolated from PCD patients. Interestingly though, PBMCs from PCD patients and Ped CO tended to produce lower amounts of IL-10 (an anti-inflammatory cytokine) compared to Ad CO (Fig. 6c). Overall, we can conclude that upon stimulation *in vitro*, PBMCs from PCD patients produce higher levels of pro-inflammatory cytokines compared to PBMCs of healthy controls. The chemokine levels produced, were high enough (10 ng/ml range) to trigger a biological response, except for CCL18 of which the maximal production reached 0.1 ng/ml (data not shown).

## Discussion

Due to ineffective mucociliary clearance in the airways, PCD patients suffer from recurrent infections in the upper and lower airways. Today, PCD patients are treated analogously to patients with CF, which is both genetically and symptomatically a different disease^12^. Our hypothesis is that besides ineffective clearance of pathogens, an affected innate immune system contributes to the recurrent airway infections in PCD patients. Monocytes are key players during infection by detecting microbial molecules, attracting and stimulating other immune cells and serving as a link to the adaptive immune system^15^. Boon *et al*.^28^ reported a link between PCD and humoral immunodeficiency disorder (HID), a rare disease characterized by hypogammaglobulinemia, defective specific antibody production and an increased susceptibility to infections. They found that in a large cohort of PCD patients, 6.5% of the patients also suffered from HID, suggesting that a common pathophysiological pathway results in both disorders^28^. These results provide a first indication of an abnormal immune system in PCD patients. In this paper, we studied the chemotactic and antibacterial properties of peripheral blood monocytes of PCD patients. To the best of our knowledge, this is the first in depth study of peripheral blood monocytes from patients with PCD.

First, we found higher C5aR expression on PBMCs of PCD patients compared to both healthy control groups. The receptors that bind the main chemokine attractants of monocytes (CCR1 for CCL3 and CCR2 for CCL2) that mediate monocyte recruitment to sites of infection were equally expressed on the monocytes of the three groups.

Chemokine and chemoattractant receptor expression are tightly regulated to prevent aberrant functioning of the innate immune system^29,30^. We did not observe altered chemotactic migration of PCD monocytes towards C5a (ligand of C5aR) compared to the healthy control groups. Neither did we measure distinctive chemotactic migration towards the other chemoattractants. Further research is necessary to elaborate if these elevated receptor expression levels have a potential role in the pathogenesis of PCD lung disease. Aberrant C5aR expression on leukocytes has been associated with several inflammatory diseases^31^.

We also examined the expression of the adhesion molecules CD15 and CD62L to investigate the capacity of monocytes from PCD patients to adhere to the endothelium. Baseline expression on non-stimulated monocytes was similar in the healthy adults, healthy children and PCD patients. Activation of monocytes leads to downregulation of selectins to allow firm adhesion on the endothelium. We observed that fMLP provoked higher CD15 upregulation and CD62L downregulation compared to CCL2. After fMLP stimulation, monocytes from PCD patients displayed reduced CD15 upregulation. Since CCL2 did not significantly alter CD15 expression on healthy monocytes, it is not clear whether moderated CD15 upregulation is a general property of PCD monocytes or is specific for their response to fMLP^32^.

Phagocytosis is a key defense mechanism of monocytes during infection and inflammation. Pathogens and cell debris can be cleared by monocytes to prevent further damage to tissues^13^. We speculated that a reduced phagocytic capacity of monocytes from PCD patients could contribute to the chronic inflammatory milieu observed in the airways of PCD patients. We assessed phagocytosis by using *Staphylococcus aureus* labeled fluorescent beads and measured intake of the beads by flow cytometry. We observed lower intake of the beads by PBMCs from PCD patients compared to Ad CO (not significant), but similarly lower uptake was also measured with PBMCs from some Ped CO. This could indicate that the capacity to phagocytose pathogens and debris by monocytes is age-dependent. Lower phagocytic capacity of monocytes from children has been detected before, although these were neonates, age 0–11 months old^33^. In the latter study, also healthy children between 1–12 years old were included and no difference in phagocytic capacity was observed compared to healthy adult controls.

To further explore the characteristics of monocytes from PCD patients, we examined the proportion of monocyte subtypes in the total monocyte population^34^. Classic monocytes (CD14++CD16−) express high levels of CCR2 and low levels of CX_3_CR1, non-classic monocytes (CD14+CD16++) lack CCR2 and express high levels of CX_3_CR1, whereas the intermediate monocytes (CD14++CD16+) express both CCR2 and CX_3_CR1. As CX_3_CR1 expression, the receptor for endothelium-bound CX_3_CL1, is higher on CD16+ and CD16++ monocytes, those subsets are thought to adhere and extravagate more efficiently compared to CD16- monocytes^35,36^. Functionally, non-classic monocytes are considered as patrollers of the vascular endothelium, both at steady state and during inflammation, and remove debris^37^. Like classic monocytes, also intermediate monocytes display pro-inflammatory characteristics, e.g. high pro-inflammatory cytokine secretion (IL-1β and TNF-α) and high MHC II expression on the cell membrane. Indeed, intermediate monocytes express significantly higher levels of TLRs 2, 4 and 5 as compared to the other two subsets. Previous studies have already described a lower CD16+ monocyte proportion in the blood circulation of children compared to adults^27^, which was confirmed in our study. The non-classic monocytes (CD14+CD16++) were significantly elevated in the blood circulation of PCD patients compared to Ped CO (p < 0.05). CD16+ and CD16++ monocyte subset increase was already observed in other inflammatory diseases, for example in patients with sepsis and rheumatoid arthritis, and is associated with disease progression^19,38^. In contrast to classic and intermediate monocytes, CD14+CD16++ cells produce only low cytokine levels under basal conditions, but respond well to TLR7/8 stimuli.

The last monocyte function examined in this study was the release of cytokines by activated monocytes, an important process in the regulation of the immune defense. We observed that stimulation with PGN provoked the most differential secretion of pro-inflammatory cytokines (significantly higher CCL3, CCL18 and CCL22 levels produced) by PBMCs from PCD patients compared to Ped CO. Also LPS, conA and IL-1β stimulation resulted in increased production of pro-inflammatory cytokines by PBMCs of PCD patients. These results suggest that in PCD patients likely more leukocytes are attracted to the site of infection through increased cytokine release by monocytes compared to age-matched Ped CO and Ad CO. This could contribute to the chronic inflammation observed in PCD patients. Indeed, increased CXCL8 sputum levels were measured in PCD patients^11^.

An advantage of this study was that we were able to include healthy pediatric controls, as there are only limited data available on pediatric values for monocyte function and chemoattractant receptor expression levels in function of age. In this way, we could demonstrate that for most functions, there is no difference between adults and children, although we confirmed some previously reported differences. Inclusion of a disease control group would better indicate whether our findings are specific for PCD. Cystic fibrosis (CF) would have been the most relevant disease control group, as both PCD and CF suffer from chronic neutrophilic inflammation in the lungs due to inefficient mucociliary clearance. However, monocytes lacking the CF transmembrane conductance regulator show reduced adhesion capacity, chemotactic migration and phagocytosis^39,40^. Consequently, this group would not be a good disease control. Patients with non-CF bronchiectasis also share some characteristics with PCD, but this group is very heterogeneous and some of these patients might suffer from an unknown underlying immune deficiency. Finally, asthma is rather an eosinophilic disorder, and has a completely different pathophysiology. Therefore, we believe that inclusion of an appropriate disease control group is not feasible.

To conclude, this study examined several characteristics of peripheral blood monocytes from PCD patients and observed differential expression of C5aR and adhesion molecules, an increased proportion of non-classical monocytes and higher production of pro-inflammatory cytokines upon stimulation. These results suggest that monocytes of patients with PCD show pro-inflammatory characteristics and can contribute to the chronic inflammation observed in PCD patients.

## Material and Methods

### Patients

We included patients between 2 and 26 years old between June 2012 and November 2016 (Table 1). Patients with an established diagnosis of PCD were recruited at the time of routine follow-up at the University of Leuven (KUL) and Université Catholique de Louvain (UCL). The diagnosis was based on the current gold standard for diagnosis of PCD: structural and functional evaluation of the cilia on a nasal or bronchial biopsy specimen, performed in the KU Leuven Laboratory of Ciliary Function^41^. All patients were clinically stable at the time of blood collection. To compare the data of PCD patients and healthy controls, both age-matched pediatric controls (Ped CO) (4–18 years, 21 individuals) and adult controls (Ad CO) (23–41 years, 19 individuals) were included. Healthy volunteers (adult personnel or their children) were recruited from the Laboratory of Molecular Immunology, the Laboratory of Immunobiology (Rega Institute, University of Leuven) and the staff of Pediatric Pulmonology. Adult controls were also included since it was impossible to process blood samples of PCD patients and age-matched pediatric controls on the same day. Blood samples from PCD and healthy controls were always taken at the most 15 minutes apart, and were processed simultaneously. In most figures, data of adult controls are equated to 100% so that the results of the pediatric controls and PCD patients could be compared to each other.

The study was approved by the Ethical Committee of the University Hospital of Leuven (S57236[ML11095]) and all patients/parents signed informed consent. All experiments were performed in accordance with relevant European guidelines and regulations.

### Reagents

Anti-CD14 [PE mouse anti-human, clone M5E2], anti-CD16 [PE mouse anti-human, clone 3G8], anti-CCR1 [Alexa Fluor 647 mouse anti-human CD191, clone 53504], anti-CCR2 [Alexa Fluor 647 mouse anti-human CD192, clone 48607], κ Isotype Control Alexa Fluor 647 [mouse IgG2b, clone 27–35], anti-BLT1 [mouse anti-human, clone 14F11], anti-FPR1 [mouse anti-human, clone 556015], anti-C5aR [mouse anti-human, clone D53–1472] and polyclonal PE goat anti-mouse were purchased from BD Biosciences (Temse, Belgium). Anti-CCR5 [mouse anti-human, clone 45531] was purchased from R&D Systems (Abingdon, UK). Anti-CD15 [FITC mouse anti-human, clone HI98] and anti-CD62L (L-selectin) [APC mouse anti-human, clone DREG-56] were obtained from eBioscience (Vienna, Austria). Anti-CD14 [APC mouse anti-human, clone M5E2] and APC anti-mouse IgG2a isotype control [clone MOPC-173] were obtained from Biolegend (San Diego, USA). LTB4 and recombinant human CCL2 were bought from Peprotech (Rocky Hill, NY), fMLP and recombinant C5a from Sigma (St. Louis, USA).

### Isolation of PBMCs

Blood samples were collected in EDTA+ tubes. PBMCs were isolated from whole blood by density gradient centrifugation with Ficoll-sodium diatrizoate (Lymfoprep, Axis-Shield PoC AS, Oslo, Norway) (400 × *g*, 30 min, 20 °C, without break). As such, PBMCs were separated from the red blood cells and neutrophils according to their density. The top layer contained both PBMCs and plasma and after collecting the PBMCs, they were washed twice with Dulbecco’s phosphate buffered saline (DPBS without Ca^2+^ and Mg^2+^; Lonza, Bazel, Switzerland) and counted in a hemocytometer.

### Flow cytometry

To measure and compare the expression of chemoattractant receptors on PBMCs of PCD patients and healthy controls, flow cytometry was used. PBMCs [3 × 10^5^ cells, diluted in FACS buffer (DPBS + 2% Fetal Bovine Serum (FBS; Life Technologies))] were stained with the following monoclonal antibodies: unlabeled anti-CCR5, anti-FPR1 and anti-C5aR; PE-labeled anti-CD16 and anti-BLT1; APC-labeled anti-CD14; Alexa 647-labeled anti-CCR1 and anti-CCR2. The cells were incubated with the antibodies for 30 min and afterwards washed 3 times with FACS buffer. Cells incubated with unlabeled antibodies were subsequently stained with goat anti-mouse PE-labeled antibody and again incubated for 30 min on ice. After 3 additional washing steps, the cells were fixed with FACS buffer containing 0.4% paraformaldehyde. Cell suspensions were applied to a FACSCalibur flow cytometer (BD Biosciences) and the results were analyzed by CellQuest software (BD Biosciences). Results represent expression on monocytes because a gate was set around the CD14-positive cells.

### Boyden microchamber chemotaxis assay

PBMC migration was measured in 48-well Boyden microchambers (Neuro Probe, Gaithersburg, MD, USA). The chemoattractants (CCL2, LTB4, fMLP and C5a) were diluted with Hanks’ balanced salt solution (HBSS; Life Technologies) containing 1 mg/ml human serum albumin (HSA; Belgian Red Cross). The lower wells of the Boyden microchambers were filled in triplicate with the diluted chemoattractants. The dilution buffer (HBSS + HSA) was used as control to assess spontaneous migration. PBMCs (2 × 10^6^ cells/ml, diluted in HBSS + HSA) were added in the upper wells, which were separated from the lower wells by a polyvinylpyrrolidone (PVP) polycarbonate membrane (5 µm pore size; GE Water & Process Technologies, Manchester, UK) and incubated at 37 °C, 5% CO_2_ for 2 h. These conditions favor monocyte migration, rather than lymphocyte migration. After incubation and treatment of the chemotaxis membrane with Hemacolor solutions (Merck, Darmstadt, Germany), the migrated cells were counted microscopically (500 x magnification) in 10 high power fields/well. The chemotactic index (CI) was calculated by dividing the average number of migrated cells in response to the chemoattractant by the average number of spontaneously migrated cells. A relative chemotactic index (CI) was used wherein the CI of the adult control tested on the same day as PCD patients or pediatric controls is equated to 100%. As such, we could compare CIs between different Boyden chambers and between pediatric controls and PCD patients.

### Adhesion molecules activity assay

The downregulation of the selectin CD62L and the upregulation of selectin ligand CD15 on monocytes (gated on CD14 positivity) after stimulation was measured by flow cytometry, as previously described by Mortier *et al*.^25^. Whole blood of patients and healthy donors was diluted to 1 × 10^6^ leukocytes/ml in warm DPBS (37 °C). Leukocytes (500 µl/tube) were then stimulated with 10^−8^ M fMLP or 300 ng/ml CCL2, both diluted in warm DPBS (37 °C). Stimulation with warm DPBS alone was used as control condition. The cells were incubated at 37 °C for 10 min. Activation was stopped by placing the cells on ice and adding 1 ml of ice-cold DPBS. After centrifugation (7 min, 1200 rpm, 4 °C), ice-cold FACS buffer was added and the cells were placed on ice for 15 min to block the Fc-receptors. Afterwards, the cells were stained with the following monoclonal antibodies, diluted in FACS buffer: PE-labeled anti-CD14, APC-labeled anti-CD62L and FITC-labeled anti-CD15. The cells were washed with FACS buffer and fixed with fixation buffer (*vide supra*) at 4 °C for 15 min. Red blood cells were removed with FACS lysing solution (BD Biosciences). Afterwards, the cells were washed once and subjected to flow cytometry (*vide supra*).

### Phagocytosis assay

The phagocytic capacity of PBMCs was assessed by flow cytometry. PBMCs were diluted in phagocytosis uptake buffer (1.5 × 10^6^ cells/ml; Live cell imaging solution, Molecular Probes, Eugene, Oregon) and *Staphylococcus aureus* labeled beads (pHrodo red *S*. *aureus* bioparticles; Molecular Probes) were added to the cells. These beads are sensitive to differences in *p*H and after uptake in acidic phagolysosomes, the fluorescence signal of the beads increases. The cells were incubated for 30 min (37 °C, 5% CO_2_) to stimulate phagocytic uptake of the beads (negative controls were incubated at 4 °C). Afterwards, the cells were washed twice with FACS buffer, fixed and evaluated by flow cytometry (*vide supra*).

### Induction of cytokine production by PBMCs

Freshly isolated PBMCs were diluted in induction medium (2 × 10^6^ c/ml; RPMI 1640 + 2% FBS + 0.01% gentamycin) and seeded in a 48-well plate. The cells were stimulated with the indicated concentrations of lipopolysaccharide (LPS; Sigma), peptidoglycan (PGN; Sigma), concanavalin A (conA), the dsRNA polyriboinosinic:polyribocytidylic acid (PIC; P-L Biochemicals, Milwaukee, WI) or recombinant human IL-1β at 37 °C and 5% CO_2_. After 24 h, the cell supernatants were collected, cleared from cells by centrifugation and stored at -20 °C. TNF-α, IL-1β, IL-10, CCL3, CCL5, CCL18 and CCL22 concentrations in the cell supernatants were determined by sandwich ELISA. The CCL3 ELISA is developed in our laboratory^42^. TNF-α, IL-1β, IL-10, CCL5, CCL18 and CCL22 DuoSet antibody pairs for ELISAs were purchased from R&D systems.

### Statistical analysis

All data are expressed in median values, unless mentioned otherwise. Comparison between groups was performed with the non-parametric Mann-Whitney U-test (Graphpad Software 6, GraphPad Software, Inc., La Jolla, USA). A p-value < 0.05 was regarded as statistically significant.

## References

[1] Boon M, Jorissen M, Proesmans M, De Boeck K (2013). Primary ciliary dyskinesia, an orphan disease. Eur J Pediatr.

[2] Knowles MR, Zariwala M, Leigh M (2016). Primary Ciliary Dyskinesia. Clin Chest Med.

[3] Werner C, Onnebrink JG, Omran H (2015). Diagnosis and management of primary ciliary dyskinesia. Cilia.

[4] Munkholm M, Mortensen J (2014). Mucociliary clearance: pathophysiological aspects. Clin Physiol Funct Imaging.

[5] Horani A, Brody SL, Ferkol TW (2014). Picking up speed: advances in the genetics of primary ciliary dyskinesia. Pediatr Res.

[6] Kurkowiak M, Zietkiewicz E, Witt M (2015). Recent advances in primary ciliary dyskinesia genetics. J Med Genet.

[7] Shapiro AJ (2016). Diagnosis, monitoring, and treatment of primary ciliary dyskinesia: PCD foundation consensus recommendations based on state of the art review. Pediatr Pulmonol.

[8] Satir P, Christensen ST (2007). Overview of structure and function of mammalian cilia. Annu Rev Physiol.

[9] Kobbernagel HE (2016). Study protocol, rationale and recruitment in a European multi-centre randomized controlled trial to determine the efficacy and safety of azithromycin maintenance therapy for 6 months in primary ciliary dyskinesia. BMC Pulm Med.

[10] Sagel SD, Chmiel JF, Konstan MW (2007). Sputum biomarkers of inflammation in cystic fibrosis lung disease. Proc Am Thorac Soc.

[11] Bush A (2006). Mucus properties in children with primary ciliary dyskinesia: comparison with cystic fibrosis. Chest.

[12] Ratjen F (2016). Changes in airway inflammation during pulmonary exacerbations in patients with cystic fibrosis and primary ciliary dyskinesia. Eur Respir J.

[13] Dale DC, Boxer L, Liles WC (2008). The phagocytes: neutrophils and monocytes. Blood.

[14] Headland SE, Norling LV (2015). The resolution of inflammation: Principles and challenges. Semin Immunol.

[15] Yona S, Jung S (2010). Monocytes: subsets, origins, fates and functions. Curr Opin Hematol.

[16] Murdoch C, Finn A (2000). Chemokine receptors and their role in inflammation and infectious diseases. Blood.

[17] Luster AD (1998). Chemokines–chemotactic cytokines that mediate inflammation. N Engl J Med.

[18] Zlotnik A, Yoshie O (2000). Chemokines: a new classification system and their role in immunity. Immunity.

[19] Ziegler-Heitbrock L (2007). The CD14+CD16+ blood monocytes: their role in infection and inflammation. J Leukoc Biol.

[20] Stansfield BK, Ingram DA (2015). Clinical significance of monocyte heterogeneity. Clin Transl Med.

[21] Yegin O (1983). Chemotaxis in childhood. Pediatr Res.

[22] Gerszten RE (1999). MCP-1 and IL-8 trigger firm adhesion of monocytes to vascular endothelium under flow conditions. Nature.

[23] Griffin JD (1990). Granulocyte-macrophage colony-stimulating factor and other cytokines regulate surface expression of the leukocyte adhesion molecule-1 on human neutrophils, monocytes, and their precursors. J Immunol.

[24] Middleton J, Patterson AM, Gardner L, Schmutz C, Ashton BA (2002). Leukocyte extravasation: chemokine transport and presentation by the endothelium. Blood.

[25] Mortier A (2010). Posttranslational modification of the NH2-terminal region of CXCL5 by proteases or peptidylarginine Deiminases (PAD) differently affects its biological activity. J Biol Chem.

[26] Nourshargh S, Alon R (2014). Leukocyte migration into inflamed tissues. Immunity.

[27] Kzhyshkowska J, Gudima A, Moganti K, Gratchev A, Orekhov A (2016). Perspectives for Monocyte/Macrophage-Based Diagnostics of Chronic Inflammation. Transfus Med Hemother.

[28] Boon M, De Boeck K, Jorissen M, Meyts I (2014). Primary ciliary dyskinesia and humoral immunodeficiency–is there a missing link?. Respir Med.

[29] Mukaida N, Harada A, Matsushima K (1998). Interleukin-8 (IL-8) and monocyte chemotactic and activating factor (MCAF/MCP-1), chemokines essentially involved in inflammatory and immune reactions. Cytokine Growth Factor Rev.

[30] Zabel BA, Rott A, Butcher EC (2015). Leukocyte chemoattractant receptors in human disease pathogenesis. Annu Rev Pathol.

[31] Lee H, Whitfeld PL, Mackay CR (2008). Receptors for complement C5a. The importance of C5aR and the enigmatic role of C5L2. Immunol Cell Biol.

[32] Dadfar E, Lundahl J, Jacobson SH (2004). Monocyte adhesion molecule expression in interstitial inflammation in patients with renal failure. Nephrol Dial Transplant.

[33] Muniz-Junqueira MI (2003). Novel microtechnique for assessment of postnatal maturation of the phagocytic function of neutrophils and monocytes. Clin Diagn Lab Immunol.

[34] Ziegler-Heitbrock L (2010). Nomenclature of monocytes and dendritic cells in blood. Blood.

[35] Mukherjee R (2015). Non-Classical monocytes display inflammatory features: Validation in Sepsis and Systemic Lupus Erythematous. Sci Rep.

[36] Old EA (2014). Monocytes expressing CX3CR1 orchestrate the development of vincristine-induced pain. J Clin Invest.

[37] Thomas G, Tacke R, Hedrick CC, Hanna RN (2015). Nonclassical patrolling monocyte function in the vasculature. Arterioscler Thromb Vasc Biol.

[38] Skrzeczynska J, Kobylarz K, Hartwich Z, Zembala M, Pryjma J (2002). CD14+CD16+ monocytes in the course of sepsis in neonates and small children: monitoring and functional studies. Scand J Immunol.

[39] Sorio C (2016). Mutations of Cystic Fibrosis Transmembrane Conductance Regulator Gene Cause a Monocyte-Selective Adhesion Deficiency. Am J Respir Crit Care Med.

[40] Van de Weert-van Leeuwen PB (2013). Optimal complement-mediated phagocytosis of Pseudomonas aeruginosa by monocytes is cystic fibrosis transmembrane conductance regulator-dependent. Am J Respir Cell Mol Biol.

[41] Bush A (2007). Primary ciliary dyskinesia: current state of the art. Arch Dis Child.

[42] Gouwy M (2015). Serum amyloid A chemoattracts immature dendritic cells and indirectly provokes monocyte chemotaxis by induction of cooperating CC and CXC chemokines. Eur J Immunol.

